# Physicians’ Mutual Benefit Association

**Published:** 1887-01

**Authors:** 


PHYSICIANS MUTUAL BENEFIT ASSOCIATION.—Received, Bennington, Indiana, of Dr. F. E, Daniel, Secretary Physicians' Mutual Benefit Association of Texas, sight draft on Swenson & Sons, of New York, for $120, in payment of death benefit due Dr. Albert Welch's heirs under provisions of certificate No. 134.

MRS. REBECCA LUCINDA WELCH.

